# Association between Serum Vitamin D Levels and *VDR* *BsmI* Polymorphism in Temporomandibular Joint Dysfunction Patients (in the Iranian Population) 

**DOI:** 10.30476/dentjods.2025.106238.2644

**Published:** 2026-06-01

**Authors:** Maryam Mostafavi, Fahimeh Rezazadeh, Mohammad Javad Fattahi, Fatemeh Tavakoli, Abbas Ghaderi

**Affiliations:** 1 Post Graduate Dept. of Orthodontic, School of Dentistry, Isfahan University of Medical Sciences, Isfahan, Iran.; 2 Oral and Dental Disease Research Center, Dept. of Oral and Maxillofacial Medicine, School of Dentistry, Shiraz University of Medical Sciences, Shiraz, Iran.; 3 Shiraz Institute for Cancer Research, School of Medicine, Shiraz University of Medical Sciences, Shiraz, Iran.; 4 University at Buffalo, Oral Biology, Surgery and Biomedical Engineering, Buffalo, New York, USA.

**Keywords:** Temporomandibular Joint Disorder, Vitamin D, Serum, Receptors, Calcitriol, Insufficiency

## Abstract

**Background:**

Multiple studies have revealed an association between vitamin D levels, genetic polymorphisms of the *VDR* (vitamin D receptor) gene, and the underlying causes of various bone and cartilage disorders.

**Purpose:**

This study aimed to investigate the association between the *BsmI* polymorphism of the *VDR* gene and serum vitamin D levels in a group of temporomandibular joint dysfunction (TMD) patients compared to healthy controls.

**Materials and Method:**

Our cross-sectional study encompassed 42 TMD patients diagnosed according to the research diagnostic criteria for temporomandibular disorders (RDC /TMD) and 41 healthy subjects. Genomic DNA was prepared, the *BsmI* variant was analyzed by PCR-RFLP, and the serum vitamin D level was measured by ELISA technique. Chi square and ANOVA test was used for analysis and statistical significance was set at *p* Value < 0.05.

**Results:**

The serum levels of vitamin D in the TMD group were significantly lower than those in the control group, with values of 43.52±18.16 ng/mL compared to 57.56±21.29 ng/mL (*p*= 0.002). The prevalence of vitamin D insufficiency was remarkably higher in the patients than in the controls, at 26.19% vs. 4.87%, respectively (*p*= 0.008). Our analysis revealed no significant differences in the genotype and allele frequencies of the *VDR* *BsmI* variant between TMD patients and controls (*p*= 0.475 and *p*= 1.000, respectively). Additionally, our study found no significant association between the genotypes of the *VDR* *BsmI* variant and vitamin D status (*p*= 0.363).

**Conclusion:**

Monitoring vitamin D levels in TMD patients is important, as deficiency may impact disease development. Further research is needed on the role of vitamin D and *VDR* gene variants in managing temporomandibular disorders.

## Introduction

Temporomandibular disorders (TMDs) are a subset of craniofacial pain conditions that include a wide range of musculoskeletal and neuromuscular problems affecting the temporomandibular joint (TMJ) and adjacent nerves and muscles [ 1 - 2 ]. Various local and systemic factors contribute to the development of TMD. It can result from jaw trauma, head or neck injuries, bruxism, arthritis in the jaw joint, jaw dislocation or fracture, and other conditions that put stress on the TMJ. Psychological factors like anxiety and depression can also contribute to its onset [ 3 - 4 ]. Studies indicate that 10-15% of adults have TMD, yet 5% look for treatment [ 5 - 7 ]. Among adolescents aged 14 to 18, the prevalence reaches around 30% [ 8 ]. The most common age range is ages of 20-40, and the prevalence is twice in women [ 3 ].

TMD is one of the primary causes of non-dental pain in the oral and maxillofacial region [ 9 ]. The most common symptom is pain around the TMJ, especially when opening and closing the mouth, typically on one side. Other symptoms include neck and shoulder pain, ear discomfort, headaches, limited jaw movement, and clicking or popping sounds when chewing or opening the mouth [ 3 , 10 ]. Diagnosis of TMD primarily relies on patient history and clinical examination findings. Most patients present with pain, limited or asymmetric jaw movement, and TMJ sounds. When clinical history and findings are unclear, imaging may be utilized [ 3 , 9 ]. 

Complex mechanisms lead to TMJ disorders. In recent years, considerable effort has been focused on the identification of these mechanisms. Bone markers like serum vitamin D levels are reliable indicators for evaluating bone metabolism and health [ 11 ]. Vitamin D plays a critical role in helping to balance calcium and maintain healthy bones [ 12 ]. It is synthesized by the skin under UV radiation and activated through enzymatic hydroxylation in the liver and kidneys. 

Vitamin D deficiency is common globally, potentially affecting up to one billion people [ 12 ]. Numerous studies highlight the role of vitamin D in oral health, and its deficiency is associated with a wide range of oral diseases [ 13 - 14 ]. Serum analysis of 25-hydroxy vitamin D is a valuable biomarker analysis of vitamin D status. In addition, the Vitamin D receptor (*VDR*) gene is a key candidate for exploring genetic factors contributing to TMJ disorders [ 12 ]. It has been shown that vitamin D levels and its receptors are associated with developing bone and cartilage diseases [ 15 ].

Recent research has highlighted that low serum vitamin D levels are linked to musculoskeletal disorders, including osteoarthritis, osteoporosis, and muscle pain [ 12 , 16 - 17 ]. Studies also suggest a possible association between vitamin D deficiency, certain gene variants (such as the vitamin D receptor gene), and an increased risk of developing TMD [ 15 , 18 - 19 ]. However, more needs to be done to fully understand the underlying genetic and molecular mechanisms involved.

The current study aims to evaluate the serum levels of vitamin D and assess the impact of *VDR* gene polymorphism in patients with temporomandibular disorders. By pointing to these variables, this research aims to assess the molecular mechanisms underlying TMD and to explore the potential of vitamin D-related markers as targets for therapeutic intervention. 

## Materials and Method

### Study population

In this cross-sectional study with ethical code “IR. SUMS.DENTAL.REC.1402.026 “, 83 participants from individuals referred to the School of Dentistry of the University of Medical Sciences of Shiraz, were selected. The case group consisted of 42 individuals who had TMD based on the inclusion criteria and did not meet the exclusion criteria, while 41 individuals comprised the healthy group as the control group. A power analysis using a medium effect size, Cohen's d= 0.5, an alpha level of 0.05, and a power of 80% showed that at least 34 participants in each group were needed.

The inclusion criteria for this study consisted of individuals diagnosed with TMD according to Research diagnostic criteria for temporomandibular disorders (RDC/TMD) [ 20 ] who provided full consent to participate. Exclusion criteria included individuals currently undergoing treatment for TMD, those taking vitamin D or calcium supplements, and individuals using medications that could affect the absorption of vitamin D and calcium, such as corticosteroids, phenobarbital, and orlistat (tetrahydrolipstatin) [ 21 ]. Additionally, individuals with inflammatory or bone-related conditions that could impact vitamin D levels, such as osteoporosis or arthritis, were also excluded from the study.

Following patients' selection, an individual sheet was prepared for each participant to collect some demographic and medical information including age, sex, associated chronic medical disorders, and any current medications. All participants underwent a thorough clinical TMJ examination. To further assess the severity of TMD, we utilized two tools including VAS for measuring pain intensity and the Helkimo index for evaluating clinical dysfunction. The VAS is a simple and effective tool that consists of a 10 cm line with "no pain" at one end and "worst imaginable pain" at the other. The Helkimo index is used to assess the severity of dysfunction through a physical examination, evaluating factors such as jaw movement, joint sounds, TMJ pain, and muscle tenderness. Scores range from 0 to 25, classifying patients into categories of: no dysfunction (Di0: 0 points), mild dysfunction (DiI: 1–4 points), moderate dysfunction (DiII: 5–9 points), and severe dysfunction (DiIII: 10–25 points).

### Genotyping 

Venous blood samples were collected from all participants and sent to the Shiraz Institute for Cancer, Research of Shiraz University of Medical Sciences for genotyping the *VDR* gene and assessing vitamin D levels. DNA was extracted from the blood samples of all the patients and healthy individuals using the DNA extraction kit (Pars Tous, Iran). The *BsmI* *VDR* gene polymorphism in all the samples was analyzed using the PCR-RFLP method. The *VDR* *BsmI* site was amplified using primers 5'-CAACCAAGACTACAAGTACCG CGTCAGTGA-3' (forward) and 5'-AACCAGCGGAA GAGGTC AAGGG-3' (reverse). PCR was performed in a volume of 25 microliters and the cyclic conditions of PCR included initial denaturation at 94°C for 3 minutes, followed by denaturation at 94°C for 30 seconds, heating at 62°C for 30 seconds, at 72°C for 1 minute for 30 cycles, and final extension at 72°C for 5 minutes. The *BsmI* restriction enzyme was applied to the PCR product overnight at 37°C. The resulting product was loaded onto a 2% agarose gel and stained with a safe stain for visualization under UV light. Three genotypes were obtained by digestion with *BsmI*: GG , G-A , and AA [BB (825 bp)], G-A [Bb (825, 650, 175 bp)], and AA [bb (650, 175 bp)]. 

### Vitamin D level

The vitamin D levels were measured in the laboratory after the clinical examination using a sensitive 25-OH vitamin D ELISA kit (Monokit, Iran, licensed by MON-OBIND, Inc.) with the standard concentrations of 25-OH vitamin D, provided by the manufacturer, and presented as ng/ml. All the samples were categorized into four groups based on vitamin D levels defined as Deficient (<20ng/mL), insufficient (20-29ng/mL), sufficient (30-100ng/mL), and potential toxicity (>100ng/ mL).

### Statistical analysis

Statistical analysis was performed by using IBM SPSS version 22.0 for Windows. The chi-square test was applied to test the association between categorical variables. Serum vitamin D level was compared in patients and healthy controls by using an independent t-test. In addition, one-way ANOVA was used to determine the differences in serum vitamin D levels at different grades of the Helkimo index. Differences in genotype and allele distributions were calculated using Pearson’s chi-square test with Yates correction. Statistical significance was set at *p*< 0.05, using two-tailed *p* values.

## Results

The sample consisted of 83 individuals: 42 (50.60%) patients and 41 (49.40%) healthy controls. Most of the participants, 52 (62.65%), were females, while 31 (37.35%) were males. Table 1 shows the demographic, clinical characteristics, and laboratory findings of the participants enrolled in the study.

**Table 1:** Demographic, laboratory findings, and clinical characteristics of the study population

Variables		Patients	Healthy Controls	*p* Value
Age (Mean ± SD)		30.40 ± 13.17	42.90 ± 7.08	*p*= 0.040
Gender	Female	30 (71.43%)	22 (53.66%)	*p*= 0.094
	Male	12 (28.57%)	19 (46.34%)	
Serum vitamin D levels (Mean ± SD)		43.52±18.16ng/mL	57.56±21.29 ng/mL	*p*= 0.002
Serum vitamin D category	Deficient (<20 ng/mL)	0 (0%)	0 0%)	*p*= 0.005
	Insufficient (20-29 ng/mL)	11 (26.2%)	2 (4.9%)	
	Sufficient (30-100 ng/mL)	31 (73.8%)	38 (92.7%)	
	potential toxicity (>100 ng/mL)	0 (0%)	1 (2.4%)	
Serum Vitamin D status	Insufficient (< 30 ng/mL)	11 (26.19%)	2 (4.87%)	*p*= 0.008
	Sufficient (>30 ng/mL)	31 (73.80%)	39 (95.12%)	
Helkimo Index (Mean ± SD)		6.31 ± 2.95	-	-
Helkimo index category	Grade 0	0 (0%)	-	-
	Grade 1 (1-4)	10 (23.80%)		
	Grade 2 (5-9)	24 (57.14%)		
	Grade 3 (+10)	8 (19.04%)		
VAS scores (Mean ± SD)		3.75 ± 2.80	-	-

VAS: visual analogue scale

The male-to-female ratio did not significantly differ between the patient and control groups (*p*= 0.094), indicating that the groups were sex-matched.

Although there was a significant age difference between the two groups (*p*= 0.04), age was included as a covariate in analysis of covariance (ANCOVA) and multiple regression modeling. The results demonstrated that the difference in vitamin D levels between patients and controls remained statistically significant even after adjusting for age, confirming that age differences did not bias the findings.

The male-to-female ratio did not significantly differ between the patient and control groups (*p*= 0.094), indicating that the groups were sex-matched.

Although there was a significant age difference between the two groups (*p*= 0.04), age was included as a covariate in ANCOVA and multiple regression modeling. The results demonstrated that the difference in Vitamin D levels between patients and controls remained statistically significant even after adjusting for age, confirming that age differences did not bias the findings.

**Figure 1:**
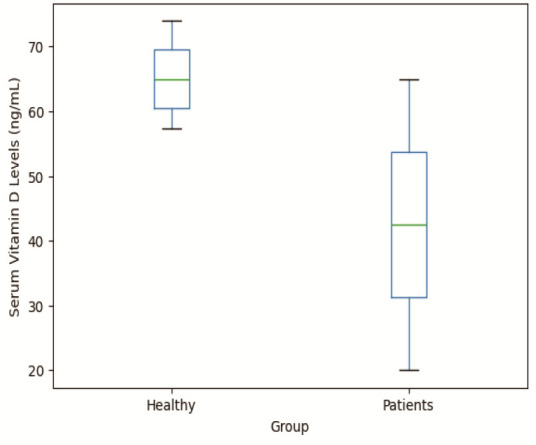
The box plot shows the changes in serum vitamin D levels in Temporomandibular Joint Dysfunction (TMD) patients compared to controls (healthy)

Figure 1 illustrates the mean serum vitamin D levels in the study groups. The control group had mean serum vitamin D levels of 57.56±21.29ng/mL, while the patient group had mean serum vitamin D levels of 43.52± 18.16ng/mL, with a statistically significant difference between them (*p*= 0.002) (Figure 1). 

Additionally, a comparison of serum vitamin D levels categorized as sufficient versus insufficient showed more insufficient levels in TMD patients compared to healthy controls (*p*= 0.008). In the analytical evaluation, due to the absence of individuals with serum vitamin D deficiency in both groups and the presence of only one individual with a potential toxicity level of vitamin D in the healthy control group, a new categorization was made to compare the serum level of vitamin D: Insufficient (<30ng/mL) and Sufficient (>30 ng/mL).

In this study, the mean VAS score for TMD patients was 3.75±2.80, indicating mild to moderate pain intensity and the mean Helkimo index score was 6.31±2.95, indicating moderate dysfunction. A detailed analysis showed that eight participants had grade 3 (severe dysfunction) and ten had grade 1 (mild dysfunction). The mean serum level of vitamin D for grades I to III of the Helkimo index was 50.80, 39.29, and 47.12 ng/mL respectively. The findings revealed no statistically significant association between serum vitamin D levels and the severity of TMD (*p*= 0.203). 

**Figure 2:**
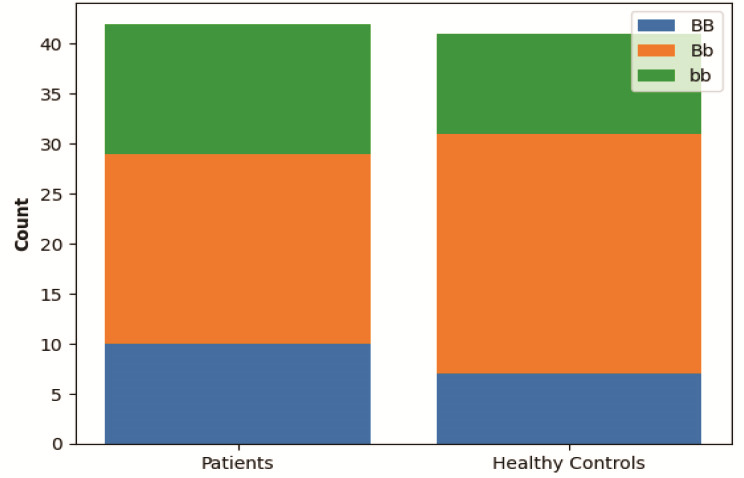
Genotype distribution of vitamin D receptor (*VDR*) *BsmI* variant in Temporomandibular Joint Dysfunction (TMD) patients vs. healthy group

The *VDR* *BsmI* variant genotype distribution was in Hardy-Weinberg equilibrium in both TMD patients and healthy controls in this study. As shown in Table 2, the frequencies of *VDR* *BsmI* variant genotypes in TMD patients were 10 (23.80%) for BB, 19 (45.23%) for Bb, and 13 (30.95%) for bb. In controls, the frequencies were 7 (16.66%) for BB, 24 (58.53%) for Bb, and 10 (24.39%) for bb, with no significant differences between the two groups (*p*= 0.475) (Figure 2). Our analysis also revealed no significant differences in the allele frequency of *VDR* *BsmI* variant between TMD patients and controls.

**Table 2:** distribution of VDR BsmI variant genotypes based on serum vitamin D level status and the Helkimo index in the study population

			Genotypes			*p* Value
			BB	Bb	bb	
Serum Vitamin D status	Patients	Insufficient	0 (0%)	1 (50%)	1 (50%)	*p*= 0.363
		Sufficient	7 (17.9%)	23 (59.0%)	9 (23.1%)	
	Healthy group	Insufficient	1 (9.1%)	5 (45.5%)	5 (45.5%)	
		Sufficient	9 (29.0%)	14 (45.2%)	8 (25.8%)	
Helkimo index	Patients	Grade 1	4 (40%)	5 (50%)	1 (10%)	*p*= 0.488
		Grade 2	5 (20.8%)	10 (41.7%)	9 (37.5%)	
		Grade 3	1 (12.5%)	4 (50%)	3 (37.5%)	

VDR: vitamin D receptor

The statistical analysis revealed no significant association between genotypes of the *VDR* *BsmI* variant and vitamin D status (*p*= 0.363). The relationship between the Helkimo index (disease severity) and different genotypes of the *VDR* *BsmI* variant was also evaluated. The results showed no statistically significant correlation between the Helkimo index and the various genotypes (*p*= 0.488) (Table 2).

## Discussion

The causes of TMD are varied and multifaceted. Predisposing factors for TMDs include occlusal anomalies, trauma, parafunctional habits, psychological stress, and systemic diseases [ 23 ]. Vitamin D is critical for the maintenance of the health of the different part of joint, including cartilage, bone, and muscles, which are essential for the proper functioning of the TMJ. These findings highlight vitamin D's potential importance in TMD pathophysiology [ 24 - 25 ]. Both environmental and genetic factors influence disease susceptibility. Genetic studies that link epidemiological data with molecular insights have garnered significant attention in osteochondral disease research. Variants in the *VDR* gene have been extensively studied for their associations with osteoarthritis across various cartilage tissues [ 25 - 27 ]. However, the results of these studies have been controversial, and no definitive conclusions have been drawn about the relationship between *VDR* polymorphisms and TMD risk. This study aimed to evaluate serum vitamin D levels and assess the impact of *VDR* gene polymorphism in patients with temporomandibular disorders.

Based on our demographic data, females with TMD were more than males, which is consistent with previous studies [ 28 - 29 ]. This gender disparity may be due to hormonal differences, higher stress levels, or sociocultural factors that predispose women to TMD [ 28 ]. Additionally, the younger generation faces increasing stressors related to education, career, and social pressures, which may lead to conditions such as TMD. These factors may explain why more female patients were involved in this study and why the patient group was younger on average [ 29 ] (Table 1).

Szulc *et al*. [ 24 ] showed that vitamin D3 may play an important direct and indirect role in the development of osteoarthritis of TMJ. Kui *et al*. [ 8 ] suggested that for patients with temporomandibular disorders who are deficient in vitamin D, vitamin D supplements may be beneficial as an independent therapy. Nemati *et al*. [ 19 ] concluded that serum levels of vitamin D are lower in patients with temporomandibular joint disorder compared to healthy controls. Our study found that TMD patients had insufficient serum vitamin D levels, whereas the control group had sufficient levels. This finding emphasizes the importance of checking and potentially supplementing vitamin D in TMD patients. However, there was no significant difference in the genotype distribution of the *VDR* *BsmI* variant between TMD patients and healthy participants (Table 2, Figure 2). 

The *VDR* may play a role in vitamin D deficiency and TMD. *VDR* gene polymorphisms have been studied in various diseases, including internal derangement of the temporomandibular joint (TMJ-ID). Research in knockout animal models has shown that the *VDR* gene encodes an intracellular receptor protein that is an important mediator of vitamin D function [ 30 - 31 ]. The *VDR* gene is located on chromosome 12 (12q13.11). One of the most significant subtypes of *VDR* gene polymorphism is the *VDR* *BsmI* polymorphism, which is located in the 3′ untranslated region (UTR) and regulates the stability of *VDR* mRNA. The *VDR* *BsmI* polymorphism (rs1544410) is associated with changes in bone mineral density and circulating levels of osteocalcin [ 31 - 32 ]. Various studies on the *VDR* *BsmI* polymorphism and osteoporosis proneness have produced inconsistent results [ 26 , 31 , 33 ]. Pouresmaeili *et al*. [ 32 ] found that the *VDR* gene *BsmI* polymorphism is significantly associated with lumbar spine bone mineral density) and may have a small effect on proximal femur bone mineral density. Colombini *et al*. [ 25 ] investigated the relationship between specific genetic variants in the *VDR* and vitamin D levels but found no significant associations. Yıldız *et al*. [ 34 ] reported that the *VDR* *BsmI* variant is not a risk factor for the development of bruxism in TMD. Patients with the bb genotype and b allele were found to have a higher risk of TMD development compared to those with the BB genotype and B allele. However, in the current study, statistical analysis revealed no significant association between the polymorphism of *VDR* *BsmI* and TMD. This indicates that the distribution of this genetic polymorphism was similar between the TMD patient group and the control group.

There are several limitations to this study, including the ability to establish causality between vitamin D levels and TMD, which is related to the cross-sectional design of this study. Longitudinal research is needed to determine whether vitamin D deficiency precedes the onset of TMD or if it is a consequence of the disorder and also assessing the potential confounding factors. Future research should focus on larger, longitudinal studies to confirm the findings of this study and to explore the contributing association between vitamin D deficiency and TMD. Additionally, investigating other genetic markers and their interactions with vitamin D metabolism could provide a more comprehensive understanding of the molecular mechanisms underlying TMD. Exploring the efficacy of vitamin D supplementation in the management of TMD symptoms could also offer valuable insights into potential therapeutic strategies.

## Conclusion

This study showed that patients with TMD exhibited significantly lower serum vitamin D levels and a higher prevalence of vitamin D insufficiency compared to healthy controls, emphasizing the need to monitor and address vitamin D insufficiency in this group of patients. The results also highlight the possible role of vitamin D levels in the pathophysiology of TMD. However, no significant association was found between the *VDR* *BsmI* polymorphism and TMD, suggesting that genetic variations in this receptor may not play a primary role in TMD susceptibility. The findings reinforce the importance of vitamin D as a potential biomarker and therapeutic target in managing TMD, though the lack of correlation between vitamin D levels and TMD severity (as measured by the Helkimo index) suggests that other factors contribute to the clinical presentation. 
